# Defining Seropositivity Thresholds for Use in Trachoma Elimination Studies

**DOI:** 10.1371/journal.pntd.0005230

**Published:** 2017-01-18

**Authors:** Stephanie J. Migchelsen, Diana L. Martin, Khamphoua Southisombath, Patrick Turyaguma, Anne Heggen, Peter Paul Rubangakene, Hassan Joof, Pateh Makalo, Gretchen Cooley, Sarah Gwyn, Anthony W. Solomon, Martin J. Holland, Paul Courtright, Rebecca Willis, Neal D. E. Alexander, David C. W. Mabey, Chrissy h. Roberts

**Affiliations:** 1 Clinical Research Department, London School of Hygiene & Tropical Medicine, London, United Kingdom; 2 Division of Parasitic Diseases and Malaria, Centers for Disease Control and Prevention, Atlanta, Georgia, United States of America; 3 Center for Ophthalmology, Ministry of Health, Vientiane, Lao People’s Democratic Republic; 4 Trachoma Control Programme, Ugandan Ministry of Health, Kampala, Uganda; 5 NTD Support Centre, Decatur, Georgia, United States of America; 6 ENVISION PROGRAMME, RTI International, Kampala, Uganda; 7 Medical Research Council Unit The Gambia, Fajara, The Gambia; 8 IHRC, Inc., Centers for Disease Control and Prevention, Atlanta, Georgia, United States of America; 9 Department of Control of Neglected Tropical Diseases, World Health Organization, Geneva, Switzerland; 10 Kilimanjaro Centre for Community Ophthalmology, University Cape Town, Cape Town, South Africa; 11 International Trachoma Initiative, The Task Force for Global Health, Decatur, Georgia, United States of America; 12 MRC Tropical Epidemiology Group, Infectious Disease Epidemiology Department, London School of Hygiene & Tropical Medicine, London, United Kingdom; Fondation Raoul Follereau, FRANCE

## Abstract

**Background:**

Efforts are underway to eliminate trachoma as a public health problem by 2020. Programmatic guidelines are based on clinical signs that correlate poorly with *Chlamydia trachomatis* (Ct) infection in post-treatment and low-endemicity settings. Age-specific seroprevalence of anti Ct Pgp3 antibodies has been proposed as an alternative indicator of the need for intervention. To standardise the use of these tools, it is necessary to develop an analytical approach that performs reproducibly both within and between studies.

**Methodology:**

Dried blood spots were collected in 2014 from children aged 1–9 years in Laos (n = 952) and Uganda (n = 2700) and from people aged 1–90 years in The Gambia (n = 1868). Anti-Pgp3 antibodies were detected by ELISA. A number of visual and statistical analytical approaches for defining serological status were compared.

**Principal Findings:**

Seroprevalence was estimated at 11.3% (Laos), 13.4% (Uganda) and 29.3% (The Gambia) by visual inspection of the inflection point. The expectation-maximisation algorithm estimated seroprevalence at 10.4% (Laos), 24.3% (Uganda) and 29.3% (The Gambia). Finite mixture model estimates were 15.6% (Laos), 17.1% (Uganda) and 26.2% (The Gambia). Receiver operating characteristic (ROC) curve analysis using a threshold calibrated against external reference specimens estimated the seroprevalence at 6.7% (Laos), 6.8% (Uganda) and 20.9% (The Gambia) when the threshold was set to optimise Youden’s J index. The ROC curve analysis was found to estimate seroprevalence at lower levels than estimates based on thresholds established using internal reference data. Thresholds defined using internal reference threshold methods did not vary substantially between population samples.

**Conclusions:**

Internally calibrated approaches to threshold specification are reproducible and consistent and thus have advantages over methods that require external calibrators. We propose that future serological analyses in trachoma use a finite mixture model or expectation-maximisation algorithm as a means of setting the threshold for ELISA data. This will facilitate standardisation and harmonisation between studies and eliminate the need to establish and maintain a global calibration standard.

## Author Summary

Trachoma is caused by the bacterium *Chlamydia trachomatis* (Ct). Individuals who have previously been infected with Ct carry specific antibodies in their blood. Recent studies have suggested that these antibodies may be a good way to estimate the intensity of transmission of this bacterium in a population. Among people who do have antibodies (seropositives) there is variation in the amount that is detectable in their blood. Some people have such low levels that differentiating them from those who don’t have antibodies (seronegatives) is challenging. We used a new test for Ct antibodies on blood specimens from three countries. Our test worked extremely well, giving reproducible results when we tested the same samples multiple times. We compared four different methods for setting the position of the threshold line between seronegatives and seropositives. The estimated transmission intensity in each country varied depending on the threshold method used, but two methods that used statistical modelling algorithms to define the two groups performed consistently across all three countries’ samples. We recommend that future studies should consider adopting the statistical modelling approaches, as they are objective tests that require no reference material and allow for standardisation between studies.

## Introduction

Trachoma is caused by ocular infection with the bacterium *Chlamydia trachomatis* (Ct) [1]. It is the leading infectious cause of blindness worldwide [2]. The World Health Organization (WHO) estimates that over 200 million people in 42 countries are at risk from trachoma blindness [3], that 1.4 million people experience moderate to severe visual impairment because of the disease and that of these, around 450,000 have been irreversibly blinded [4].

The most commonly used system for estimating the prevalence of trachoma uses the WHO simplified grading system [5] of clinical signs of trachoma. These include trachomatous inflammation—follicular (TF), trachomatous inflammation—intense (TI) and trachomatous trichiasis (TT), which is the rubbing of the eyelashes against the globe of the eye. WHO guidelines recommend the SAFE strategy to combat trachoma: Surgery to treat trichiasis, annual mass-drug administration (MDA) of Antibiotics to treat Ct infection and Facial cleanliness and Environmental improvement to reduce transmission. Implementation of the SAFE strategy and cessation of MDA depends on the prevalence of TF in children aged 1–9 years. Concerns have been raised about the appropriateness of having treatment guidelines based on clinical signs such as TF and TI. In some low endemicity [6,7] and post-MDA settings [8,9], both TF and TI correlate poorly with the prevalence of Ct infection and both clinical signs are sometimes associated with bacteria other than Ct [10,11].

Tests for infection have been suggested as possible tools for trachoma control programmes. Numerous nucleic acid-amplification tests (NAATs) have been developed, including the adapted use of commercial kits originally designed for diagnosing genitourinary Ct infections [12–16]. NAATs have been shown to be cost-effective in some settings [17] but concerns have been raised that the per-sample cost of NAATs can be too much for national eye health programmes in countries where trachoma remains a problem [18]. The cost of specialist devices and platforms for deploying NAATs can also be prohibitive.

Serology has been suggested as a possible alternative to clinical signs and infection testing, as it indicates the cumulative exposure to Ct [19,20], with the potential to assess the impact of intervention efforts [21]. By monitoring the exposure to Ct of the youngest age groups, born after implementation of MDA, serology may prove useful for confirming that transmission has been interrupted [22].

Serology has recently been used in several studies [19,20,22–24], three of which have taken place in districts that have completed three or more rounds of MDA [22–24]. These studies have used the multiplex bead array platform (Bio-rad, Hercules, California) to detect antibodies against Pgp3 and CT694, antigens thought to be highly immunogenic [25]. Because this platform is costly, technically complex and unlikely to be found in most laboratories in resource-limited regions, alternative, simpler methods of antibody detection have been proposed [22,26].

To make serological testing more widely accessible, the Pgp3/CT694 assay used in previous studies [19,20,22–24] has been adapted for use in a simple Pgp3-specific enzyme-linked immunosorbent assay (ELISA). Pgp3 is a Ct-specific 84kDa heterotrimeric protein [27] and is recognised by specific IgG [28]. It is thought to be the most immunodominant of the proteins encoded by the Ct plasmid that is unique to Ct [29].

ELISAs are routinely used to detect specific IgG in dried blood spots [30–34]. ELISA data, measured as optical density (OD) is quantitative and continuous. It is desirable to be able to assign a classification (seronegative, seropositive) to each sample, but this can be challenging because the distributions of OD values in the negative and positive populations may overlap to a greater or lesser extent [34]. The aim of this study was to determine the most appropriate method for setting the threshold for positivity as well as to determine the usefulness of an anti-Pgp3-specific ELISA for identifying communities in which the transmission of ocular Ct has been interrupted. We tested dried blood spots collected as part of trachoma surveys in three countries: Laos, Uganda and The Gambia. We evaluated the age-specific seroprevalence using four methods and compared the resulting estimates of prevalence of seropositivity based on six possible thresholds. We discuss the merits of the different methods in the context of programmes seeking to monitor the elimination of trachoma as a public health problem.

## Methods and Materials

### Ethics statement

This study was conducted in accordance with the Declaration of Helsinki. This study received approval from the Ethics Committee of the London School of Hygiene & Tropical Medicine (LSHTM; references 6319, 6514, 8355, 8918), UK; the Ministry of Health of the Lao People’s Democratic Republic (No:48 NIOPH/NECHR), Ugandan Ministry of Health (VCD-IRC/053) and The Gambia government/Medical Research Council (MRC) Joint Ethics Committee (SCC1408v2). In all countries, a local health official explained the study to each head of household, answered any questions and explained the written consent form before requesting their agreement and signature. Written (thumbprint or signature) consent was obtained from each participant or the parent or guardian of each child under 18 who participated; assent was sought from children aged 12–17.

### Clinical assessment

Trachoma graders were trained according to the Global Trachoma Mapping Project (GTMP) protocols and were required to score a minimum kappa of 0.7 for the diagnosis of TF in an inter-grader agreement test with 50 eyes of 50 children [35,36]. The samples in Laos were collected in November 2014 as part of a follow-up study to the GTMP work completed there. Three districts in three regions were selected based on baseline trachoma survey findings that indicated potential ‘hot spots’ [37]. From these three regions, all children aged 1–9 in selected villages were invited to participate. Trachoma elimination programmes have never been undertaken in Laos. In Uganda, samples were collected as part of a trachoma impact survey in May 2014, following three years (2010–2012) of implementation of the A, F and E components of the SAFE strategy in two regions (Pader and Agogo). Prior to MDA, trachoma was considered highly endemic in these regions, although no data is publicly available. This study was a population based prevalence survey, which used a two stage sampling strategy; villages were selected with probability proportional to size, and households were randomly selected within each selected village based on a household list produced by the village chief and local health officials. All children aged 1–9 years in the selected households were invited to participate. In The Gambia, a population based prevalence survey using a two stage sampling strategy was undertaken in February-March 2014; villages were selected with probability proportional to size, and households were randomly selected within each selected village based on a household list produced by the village chief and local health officials. One region, Lower River Region (LRR) had undergone three rounds of annual (2007–2009) MDA for trachoma, while the other, Upper River Region (URR), has never had trachoma elimination activities because trachoma has not been of a sufficiently high prevalence to justify implementation. All members of randomly selected households were invited to participate, regardless of age.

After informed consent was obtained, a trachoma grader examined both eyes for signs of trachoma using a binocular loupe (2.5×) and a torch. The grader changed gloves between each participant to minimise the risk of carry-over contamination. Antibiotics were provided to individuals with evidence of active trachoma and/or the affected household, according to each country’s national policy.

### Blood collection

Each participant had a finger-prick blood sample collected onto filter paper (Trop-Bio Pty, Townsville, Australia), using a sterile single-use lancet (BD Microtrainer, Dublin, Ireland). Each filter paper had six extensions, calibrated to absorb 10 μL of blood. Samples were air-dried for approximately five hours and then stored in individual Whirl-Pak plastic bags (Nasco, Modesto, California) with desiccant sachets (Whatman, Little Chalfont, UK) before being stored at -20°C.

All samples were shipped to LSHTM for testing.

### ELISA analysis of anti-Ct-Pgp3 antibodies

Dried blood spots (DBS) were tested for antibodies against Pgp3. One whole filter paper extension per sample was eluted in 250 μL PBS + 0.3% v/v Tween-20 (PBSTw) (Sigma-Aldrich, Dorset, UK)+ 5% w/v non-fat milk powder (PBSTw-milk) (AppliChem, Maryland Heights, USA) overnight at 4°C. Immulon 2HB 96-well plates (VWR International, Lutterworth, UK) were coated with recombinant Pgp3 protein [19] overnight at 4°C (25ng per well in 0.1M sodium carbonate buffer, pH 9.6). Plates were washed with PBSTw to remove unbound protein, blocked with 100 μL PBSTw for 1 hour at 4°C and washed two times. Control sera with known ratios of Pgp3 antibodies (1000 units, 500 units, 200 units, 50 units and negative control serum) and a blank consisting of PBSTw-milk were run on every plate. All samples and controls were tested in triplicate at a 1:50 dilution in PBSTw-milk. After 2 hours incubation on an orbital shaker at room temperature, wells were washed 5 times and 50 μL of an HRP-labelled mouse anti-human IgG(Fc)-HRP (Southern Biotech, Birmingham, USA) diluted 1:32,000 was added. Plates were incubated for 1 hour on an orbital plate shaker at room temperature then washed 5 times to remove unbound antibody. Fifty microliters of TMB (KPL, Gaithersburg, USA) was added and the mixture was incubated in the dark for 9 minutes at room temperature. The reaction was stopped with 50 μL 1N H_2_SO_4_ and optical density was read at 450 nm (OD_450_) on a Spectramax M3 plate reader (Molecular Devices, Wokingham UK). Readings were corrected for background by subtracting the average absorbance of three blank wells containing no serum, using Softmax Pro5 software (Molecular Devices, Wokingham UK).

### Data analysis

Blanked OD_450_ values for samples and controls were normalised by dividing the mean of the three wells against the mean of 200 unit control included on each plate. This was done for each plate.

Data analysis for ELISA was performed separately and masked to the results of demographic and clinical information. Statistical analysis was carried out using R [38].

### Defining seropositivity

We used four different methods for establishing a threshold for seropositivity: visual inspection of the inflection point (VIP), a finite mixture model (FMM) [39], the expectation-maximisation algorithm (EM) [40] and an receiver operating characteristic (ROC) curve based on previously-assayed dried blood spots from children in Tanzania [19]. There are as yet no accepted guidelines as to what level of sensitivity or specificity is required of a serological test; thus we referred to a previously published template [18] and established three possible thresholds from the ROC curve: one maximising specificity, one with a sensitivity greater than 80% [18] and one optimising the balance between sensitivity and specificity, by maximising Youden’s J-index [41].

### Visual inflection point (VIP)

We asked 12 arbitrarily selected non-laboratory staff and students at LSHTM to visually examine a simple plot of the sorted OD_450_ data curves and determine the inflection point for each sample set. For this exercise, we defined the inflection point as the point on the data curve where the curve changes from predominantly horizontal to predominantly vertical. The 12 values were then averaged to determine the threshold and standard deviations (SDs) were calculated.

### Finite mixture model (FMM)

A finite mixture model [42] was used to classify the samples as seropositive or seronegative based on normalised OD_450_ values. The data were fitted using maximum likelihood methods, estimating the distribution parameters for each classification group (seropositive or seronegative) as well as the proportion of samples in each category to fit the overall distribution of results [34,43,44]. The threshold for seropositivity was then defined as the mean of the Gaussian distribution of the seronegative population plus three SDs of the seronegative population [44,45]. FMM was performed on each set of samples, based on country of origin.

### Expectation-maximisation algorithm (EM)

The expectation-maximisation algorithm is similar to FMM in that it classifies samples based on population parameters. It relies on the Bayesian information criterion to select an appropriate model. EM is an iterative optimization method to estimate some unknown parameter [40], in this case the threshold between seropositive and seronegative, given the number of clusters and the normalised OD_450_ values. EM estimates where to set the threshold while maximising the likelihood of each sample parameter [40]. Using the ‘mclust’ package in R, parameters were set to specify a univariate model with equal variance between 2 clusters [46].

### Receiver operating characteristics (ROC) Curve

Serum samples from 122 children from the United States and blood spots from 11 Ct-specific PCR-positive children from Tanzania were used to make the original ROC curve [19]. A second set of 124 Tanzanian dried blood spots were assayed using the multiplex bead array and dichotomised based on the original threshold. These samples were then re-tested with the ELISA and the data from this assay were used to generate the ROC curve used in this manuscript. The R package ‘Epi’ [47] was used to generate three different thresholds: the first of which maximises Youden’s J-index to balance sensitivity and specificity [41], the second and third were set for high sensitivity (minimum 80%) and high specificity (minimum 98%), respectively.

### Statistical analysis

The prevalence of signs of trachoma and the exact binomial confidence intervals were calculated using the R ‘Stats’ package [38]. Due to the low prevalence of clinical signs, Fisher’s exact test was used to test for association [48].

Seroprevalence in each population was calculated using each of six thresholds. We also examined the relationship between the clinical data and serological data. Due to the low prevalence of clinical signs, data for clinical signs were pooled across all three studies.

## Results

### Clinical assessment

We recruited 978 Laotian children aged 1–9 years from the provinces of Attapu (n = 406), Houaphan (n = 307) and Phôngsali (n = 239). Twenty-six participants had incomplete clinical records and were excluded from further study. The proportions of the sample populations who were male were 52.9%, 60.3% and 54.0% in Attapu, Houaphan and Phôngsali, respectively. The median age was five years in all three provinces. Fifteen cases of TF were diagnosed (1.6%, exact binomial CI = 0.9%-2.6%), 11 of which were bilateral cases (Table 1). No cases of TI were observed. There was a higher prevalence of TF in Attapu (2.7%, 11/406) than in either Houaphan (1.0%, 3/307) or Phôngsali (0.4%, 1/239), (p = 0.02) using Fisher’s exact test [49] with the Simes-Bonferroni correction for multiple tests [50].

**Table 1:** Distribution of participants in three trachoma studies, including clinical signs.

Country	Province	N	TF	TI	TS	TT	CO
Laos*		952	15 (1.6%)	-	-	-	-
	Attapu	406 (42.6%)	11 (2.7%)	-	-	-	-
	Houaphan	307 (32.2%)	3 (1.0%)	-	-	-	-
	Phôngsali	239 (25.1%)	1 (0.4%)	-	-	-	-
Uganda*		2700	93 (3.4%)	8 (0.3%)	-	-	-
	Agogo	1353 (50.1%)	43 (3.2%)	1 (0.1%)	-	-	-
	Pader	1347 (49.9%)	50 (3.7%)	7 (0.5%)	-	-	-
The Gambia*		1868	30 (1.6%)	4 (0.2%)	78 (4.2%)	8 (0.4%)	1 (0.1%)
All	LRR	1028 (55.0%)	18 (1.8%)	4 (0.4%)	55 (5.4%)	7 (0.7%)	1 (0.1%)
	URR	840 (45.0%)	12 (1.4%)	0 (0.0%)	23 (2.7%)	1 (0.1%)	0 (0.0%)
1–9 year olds	LRR	383 (20.5%)	14 (3.7%)	2 (0.5%)	1 (0.3%)	0 (0.0%)	0 (0.0%)
	URR	359 (19.2%)	11 (3.1%)	0 (0.0%)	6 (1.7%)	0 (0.0%)	0 (0.0%)
≥10 year olds	LRR	645 (34.5%)	4 (0.6%)	2 (0.3%)	54 (8.45)	7 (1.1%)	1 (0.2%)
	URR	481 (25.7%)	1 (0.2%)	0 (0.0%)	17 (2.6%)	1 (0.2%)	0 (0.0%)

‘*’ Age range in Laotian and Ugandan participants was 1–9 years; age range in all Gambian participants was 1–90 years; ‘-’ not assessed.

N = Normal; F = trachomatous inflammation, follicular; TI = trachomatous inflammation-intense; TS = trachomatous scarring; TT = trachomatous trichiasis; CO = corneal opacity.LRR = Lower River Region; URR = Upper River Region.

2738 children aged 1–9 years were recruited in the Ugandan districts of Agogo (n = 1388, 49.7% male) and Pader (n = 1377, 50.4% male). 38 participants were missing complete clinical data and were excluded from further study. The median age was five years in both districts. 93 cases of TF were diagnosed (3.4%, exact binomial CI = 2.8%-4.2%), 44 of which were bilateral. Eight cases of TI were diagnosed (0.3%, exact binomial CI = 0.1%-0.6%) (Table 1). No other clinical signs were assessed. The prevalence of TF was 3.2% in Agogo and 3.7% in Pader. There was no significant difference between the estimated prevalence of TF in the two districts (TF: Χ^2^ = 0.429, p = 0.5125; TI: Χ^2^ = 3.1566, p = 0.07562).

In the Gambia we recruited participants of all ages from the Lower River Region (LRR, n = 1028, 41.9% male) and Upper River Region (URR, n = 840, 42.5% male). Ten participants were excluded from the study because they either declined to provide a blood sample (n = 1) or had incomplete clinical data (n = 9). The median age in LRR was 13 years (range: 1–88) and 11 years in URR (range: 1–90). Overall, 30 cases of TF were diagnosed (1.6%, exact binomial CI = 1.1%-2.3%), 19 of which were bilateral (Table 1). There were 25 cases of TF in children aged 1–9 years. Four cases of TI were observed (0.2%, exact binomial CI = 0.06%-0.6%), two of which were in children aged 1–9 years. Examiners found 78 cases of TS (4.2%, exact binomial CI = 3.3%-5.2%), eight cases of TT (0.4%, exact binomial CI = 0.2%-0.8%) and one case of CO (0.05%, exact binomial CI = 0.001%-0.3%). There was a significant difference in TS prevalence between the URR and LRR (Χ^2^ = 7.2435, p = 0.007116); the difference in TF prevalence was non-significant (Χ^2^ = 0.1343, p = 0.714). The prevalence of TI, TT and CO in this population was too low for meaningful statistical analysis.

Observed frequencies of clinical signs of trachoma in the various samples are summarised in Table 1. A more detailed description, including prevalence by age and gender, is presented in Supplementary S1, S2 and S3 Tables.

### Serological analysis

The five serum controls were tested in triplicate and the mean values for each plate were tracked across each sample set. The coefficient of variation was less than 10% in each of the three replicates of each control specimen. Inter-plate variation of controls was less than 15% across all plates in each sample set as shown in Table 2. A plate was permitted to have no more than one control with >15% variation from the sample set mean for that control; if a plate had two or more controls with values more than 15% greater or lesser than the sample set mean, the plate was re-run. Less than 5% of plates were re-run due to this. Table 2 shows the mean values and the accepted 15% range for the five controls.

**Table 2:** The mean OD_450_ value for the five controls sera used on the ELISA plates. Mean, SD and coefficient of variation for the five serum standards run alongside the Ugandan samples across 24 plates. Data were similar for the standards run alongside the Laotian and Gambian samples.

Control serum	Mean	SD	Coefficient of variation	Upper limit (mean+15%)	Lower limit (mean-15%)
1000u	2.01 OD_450_	0.13 OD_450_	6.47%	2.26 OD_450_	1.75 OD_450_
500u	1.74 OD_450_	0.13 OD_450_	7.38%	2.00 OD_450_	1.49 OD_450_
200u	1.11 OD_450_	0.10 OD_450_	9.46%	1.31 OD_450_	0.90 OD_450_
50u	0.63 OD_450_	0.06 OD_450_	9.45%	0.74 OD_450_	0.51 OD_450_
Negative control serum	0.28 OD_450_	0.02 OD_450_	8.51%	0.32 OD_450_	0.23 OD_450_

**Fig 1:**
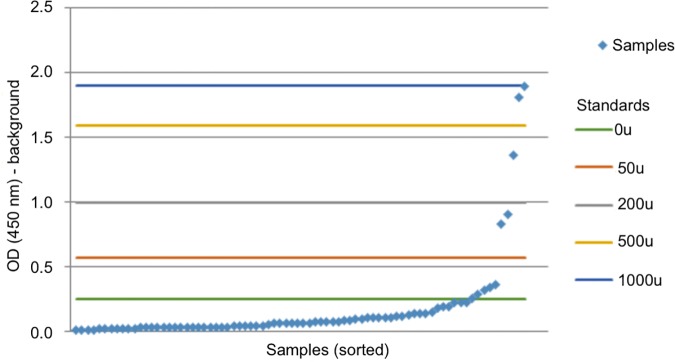
Typical results from an ELISA plate. Specimens are sorted by increasing OD values and are each represented by a separate diamond. The mean values of the controls tested in triplicate are represented by coloured horizontal lines.

**Fig 2:**
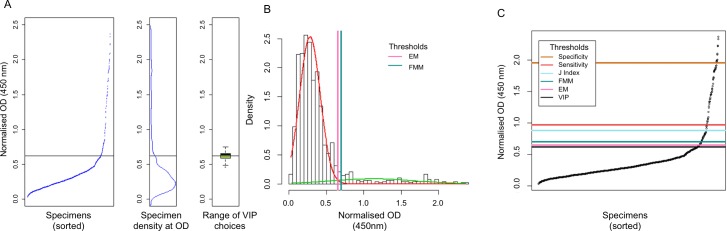
Threshold values for Laos (1–9 year olds) data. **Panel A** shows the threshold as determined by visual inflection point analysis by 12 volunteer individuals. Volunteers had access only to the data presented in the leftmost panels, which shows sorted OD_450_ values. The second panel in A shows the density of data points for the sample while the third panel in A shows a box and whisker plots with the range of threshold values that were selected by the 12 volunteers. The box shows the inter-quartile range for the values, with the thick horizontal line marking the median value. Whiskers show the upper quartile plus 1.5x the range between the 1^st^ and 3^rd^ quartiles. Outliers are shown by an open circle. **Panel B** shows the thresholds set by the finite mixture model and expectation-maximisation algorithm. Density plots of normalised OD values and thresholds, showing the FMM estimated distribution functions of ‘seronegative’ specimens in red and ‘seropositive’ specimens in green. Vertical lines show the threshold values determined by the finite mixture model (right-most line) and the expectation-maximisation algorithm (left-most lines). **Panel C** compares the threshold specifications by four different methods. Scatterplots show the normalised and sorted OD_450_ values with horizontal lines marking the thresholds specified by VIP (OD_450_ = 0.619), EM (OD_450_ = 0.650), FMM (OD_450_ = 0.696), ROC curve maximising Youden’s J-index (OD_450_ = 0.870), ROC curve with sensitivity >80% (OD_450_ = 0.968) and ROC curve with specificity>98% (OD_450_ = 1.951).

**Fig 3:**
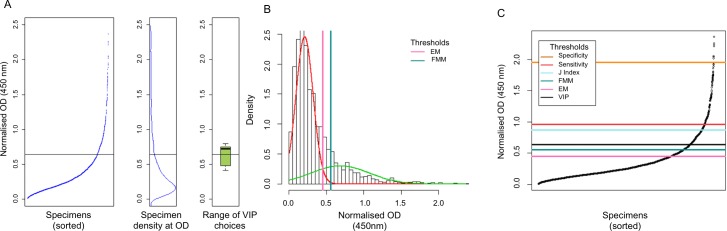
Threshold values for Uganda (1–9 year olds) data. **Panel A** shows the threshold as determined by visual inflection point analysis by 12 volunteer individuals, as detailed in Fig 2. **Panel B** shows the thresholds set by the finite mixture model and expectation-maximisation algorithm, as described in Fig 2. **Panel C** compares the threshold specifications by four different methods. Scatterplots show the normalised and sorted OD_450_ values with horizontal lines marking the thresholds specified by VIP (OD_450_ = 0.641), EM (OD_450_ = 0.450), FMM (OD_450_ = 0.554), ROC curve maximising Youden’s J-index (OD_450_ = 0.870), ROC curve with sensitivity >80% (OD_450_ = 0.968) and ROC curve with specificity>98% (OD_450_ = 1.951).

**Fig 4:**
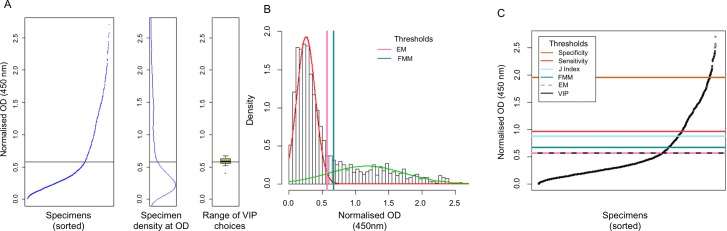
Threshold values for Gambian (all ages) data. **Panel A** shows the threshold as determined by visual inflection point analysis by 12 volunteer individuals, as detailed above in Fig 2. **Panel B** shows the thresholds set by the finite mixture model and expectation-maximisation algorithm, as described in Fig 2. **Panel C** compares the threshold specifications by four different methods. Scatterplots show the normalised and sorted OD_450_ values with horizontal lines marking the thresholds specified by VIP (OD_450_ = 0.570), EM (OD_450_ = 0.570), FMM (OD_450_ = 0.672), ROC curve maximising Youden’s J-index (OD_450_ = 0.870), ROC curve with sensitivity >80% (OD_450_ = 0.968) and ROC curve with specificity>98% (OD_450_ = 1.951). Note that the thresholds set by VIP and EM are identical (0.570 OD_450_) and overlap on the graph.

The sample set for each country was tested separately. Each plate showed a large but narrowly distributed proportion of low-OD specimens, with a smaller proportion of higher-OD specimens. Fig 1 shows typical results from an ELISA plate. In all three sample sets, density data peak around 0.25 OD_450_; this can be seen in centre panels B in Figs 2, 3 and 4.

### Visual inflection point (VIP)

The leftmost panels of Figs 2A, 3A and 4A were shown to 12 people, each of whom was asked to determine each graph’s point of inflection. The mean of the inflection points was calculated for each sample set and the SD and range were calculated. For Laos, the mean threshold was calculated to be 0.619 OD_450_ (SD = 8.2%, range 0.485–0.750); for Uganda the threshold was calculated to be 0.641 OD_450_ (SD = 14.4%, range 0.410–0.795) and for The Gambia the threshold was calculated to be 0.579 OD_450_ (SD = 7.3%, range 0.402–0.673). The sorted normalised OD_450_ values are shown in Figs 2A, 3A and 4A (leftmost panels), alongside marginal density distribution plots of the same values (centre panels) and boxplots (rightmost panels) showing the range of the 12 threshold values that were selected by the volunteers.

### Finite mixture model (FMM)

A finite mixture model was tested on all three sample sets, setting the threshold at the mean of the seronegative population plus three SDs [44,45]. The thresholds were set at 0.6963 OD_450_, 0.5537 OD_450_ and 0.6725 OD_450_ for Laos, Uganda and The Gambia, respectively. The FMMs are shown in Figs 2B, 3B and 4B.

### Expectation-maximisation algorithm (EM)

An EM model was fitted to all three sample sets, specifying parameters for a univariate model with equal variance between 2 clusters [45]. The thresholds were set at 0.65 OD_450_, 0.45 OD_450_ and 0.57 OD_450_ for Laos, Uganda and The Gambia, respectively. The EM-derived threshold selections are shown in Figs 2B, 3B and 4B.

### ROC curve

**Fig 5:**
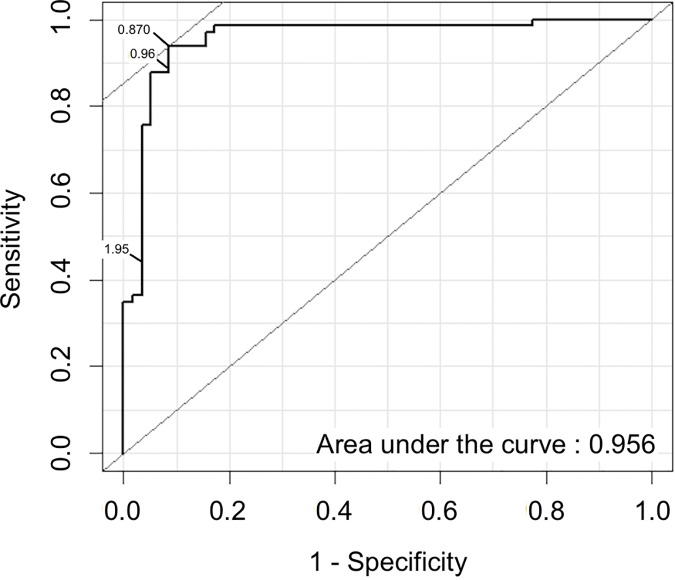
Receiver Operating Characteristic (ROC) curve showing the relationship between sensitivity, specificity and threshold values. Three different thresholds were specified to meet the requirements of: (A) an assay (threshold = 0.870 OD_450,_ specificity = 93.9%, sensitivity = 91.4%, PPV = 89.8%, NPV = 92.4%) with balanced sensitivity and specificity (maximal Youden’s J value); (B) an assay (threshold = 0.965 OD_450,_ specificity 94.8%, sensitivity = 89.4%) with at least 80% sensitivity and (C) an assay (threshold = 1.951 OD_450_, specificity = 98.3%, sensitivity = 43.9%, PPV = 66.7%, NPV = 95.0%) with at least 98% specificity.

Using the ROC curve to set a threshold optimising Youden’s J-index to balance specificity and sensitivity resulted in a threshold at 0.870 OD_450_ (specificity 93.9%, sensitivity 91.4%). Setting the threshold to ensure a minimum sensitivity of 80% resulted in a threshold at 0.965 OD_450_ (specificity 94.8%, sensitivity 89.4%). Setting the threshold for a minimum specificity of 98% resulted in a threshold at 1.951 OD_450_ (specificity 98.28%, sensitivity 43.94%). Fig 5 shows the ROC curve with the three thresholds identified.

Panels 2C, 3C and 4C show all six thresholds in relation to the normalised OD_450_ data in each of the three populations. The internally calibrated methods (i.e., VIP, FMM and EM) were reasonably conformant and appeared to favour threshold placements that were substantially lower than those set by the ROC, which is calibrated with Tanzanian specimens, even when a higher sensitivity (i.e., lower threshold value) test was specified in the ROC analysis. As a consequence of this, the seroprevalence estimates that were determined by VIP, EM and FMM were similar to one another, while the seroprevalence estimates set by any of the ROC curve thresholds were much lower in all three populations (Table 3).

**Table 3:** Seroprevalence by Country, as estimated using alternate threshold specification methods.

			Threshold % (95% Confidence Interval)										
		VIP		EM		FMM		ROC Youden’s J-index		ROC Sensitivity >80%		ROC Specificity > 98%	
	N	OD = 0.619		OD = 0.65		OD = 0.696		OD = 0.870		OD = 0.965		OD = 1.951	
**Laos (1–9 year olds)**	952	11.3%	(9.4–13.6)	10.4%	(8.6–12.6)	15.6%	(13.3–18.0)	6.7%	(5.3–8.6)	6.3%	(4.9–8.1)	1.1%	(0.5–2.0)
** **		**OD = 0.641**		**OD = 0.45**		**OD = 0.5537**							
**Uganda (1–9 year olds)**	2700	13.4%	(12.1–14.7)	24.3%	(22.7–26.0)	17.1%	(16.0–18.9)	6.8%	(5.9–7.8)	5.3%	(4.5–6.2)	0.3%	(0.1–0.8)
** **		**OD = 0.57**		**OD = 0.57**		**OD = 0.672**							
**The Gambia (all ages)**	1868	29.3%	(27.3–31.5)	29.3%	(27.3–31.5)	26.2%	(24.2–28.2)	20.9%	(19.1–22.9)	18.9%	(17.2–20.8)	3.3%	(2.6–4.3)

Seroprevalence by Gender, Region and Age is provided in Supplementary S2 Table.

VIP = visual inflection point; EM = expectation-maximisation algorithm; FMM = finite mixture model; ROC = receiver-operating characteristic curve.

OD = optical density, measured at 450 nm.

Seroprevalence for each sample set, using the six different thresholds were calculated, along with 95% confidence intervals. As the threshold increases in value, fewer specimens are classified as being seropositive, decreasing the seroprevalence. The seroprevalence for each sample set at each threshold is presented in Table 3. Seroprevalence for each country by sex, region and age is provided in Supplementary S4, S5 and S6 Tables.

Table 4 presents the proportion of seropositive samples by clinical grade, as estimated by each threshold specification. Due to the relatively low prevalence of all clinical signs, prevalence values for have been pooled.

**Table 4:** Proportion of participants with different phenotypes considered seropositive by each threshold.

	No sign of trachoma according to the WHO simplified system				Active trachoma		Scarring trachoma	
	% (95% confidence interval)				(TF and/or TI)		(TS and/or TT and/or CO)	
	1–9 year-olds		10+ year-olds		% (95%CI)		% (95%CI)	
N across all studies	4268		964		150		87	
VIP*	11.1	(10.2–12.1)	42.8	(39.7–46.0)	9.3	(5.4–15.5)	70.1	(59.2–79.2)
EM*	17.1	(16.0–18.2)	42.8	(39.7–46.0)	12	(7.5–18.6)	70.1	(59.2–79.2)
FMM*	12.7	(11.8–13.8)	38.5	(35.4–41.6)	16.7	(11.3–23.8)	69	(58.0–78.2)
J-index	5.8	(5.1–6.5)	31.8	(28.9–34.9)	4	(1.6–8.9)	55.2	(44.2–65.7)
Sensitivity >80%	4.7	(4.1–5.4)	28.8	(26.0–31.8)	3.3	(1.2–8.0)	49.4	(38.6–60.2)
Specificity >98%	0.4	(0.2–0.7)	4.8	(3.6–6.4)	0	(0–3.1)	16.1	(9.4–25.9)

*Note that country-specific thresholds were used for VIP, EM and FMM.

TF = trachomatous inflammation, follicular; TI = trachomatous inflammation-intense; TS = trachomatous scarring; TT = trachomatous trichiasis; CO = corneal opacity.

VIP = visual inflection point; EM = expectation-maximisation algorithm; FMM = finite mixture model.

## Discussion

Several previous studies have used anti-Pgp3-specific ELISAs to test for genital chlamydial infection [21,51–54] but only one [55] has used the method for the detection of antibodies against ocular chlamydial infection. In this study, we used an ELISA test to detect IgG antibodies specific to the Ct protein Pgp3 in studies with large sample sizes from three countries. To date, this is the largest study to measure antibodies to Ct in trachoma-endemic populations and the first to look at populations from more than one country, including East Africa, West Africa and Southeast Asia. We have shown that within and between runs there is a low coefficient of variation in the assay and that the bimodal data distribution of normalised OD_450_ values in those samples reflects that which would be expected in populations where a minority of individuals are seropositive and where there is a broad range of antibody titres in the seropositive sub-population. This is best observed in the data from the Gambia (Fig 4), where we included adults in the sample and where the more substantial seropositive sub-population can be accounted for by both sexually transmitted Ct infection and the formerly high level of endemicity of trachoma in the Gambia.

Clinical specimens without any Ct-specific IgG still have some degree of baseline reactivity in ELISA tests because of non-specific binding of irrelevant antibodies. There is also substantial between-specimen variation in seropositives, which reflects natural variation in the antibody titre. The potential for there being substantial overlap between the seronegative specimens with high baselines and the seropositives with low anti-Pgp3 antibody titres means that it can be difficult to differentiate between the two groups.

There is very little published information on the prevalence of trachoma in Laos and Uganda [56], but on the evidence of our analysis, clinical signs of disease are rare and the levels of seropositivity appear to be comparable to those in The Gambia, where elimination has been declared. We have no data on the prevalence of Ct infection in the communities in Laos and Uganda, nor is there any longitudinal data to monitor changes in antibody levels following documented infection. Numerous studies have looked at the prevalence of ocular Ct infection in The Gambia and shown it to be negligible [7,57,58]. All the populations we studied have received MDA and we did not screen a population with higher prevalence levels. Further research in meso- and hyper-endemic populations will be needed in order to assess the utility of this method in other settings.

We have shown how the method that is selected for the statistical interpretation of ELISA data (with particular regard to the method of threshold specification) can greatly change the population prevalence estimates that are derived. Methods that indicate the use of a higher threshold value are likely to be more specific and have a higher positive predictive value (PPV), but they do incur a penalty in the form of reduced sensitivity. In the context of post-MDA trachoma control, a test with high PPV is more desirable as over-diagnosis might lead to the inappropriate continuation of MDA interventions. Meanwhile a lower sensitivity test, applied in a low prevalence setting such as the post-MDA population of the Gambia, is likely to have a high negative predictive value (NPV) and the clinical impact of the false negative rate is likely to be modest as long as the sensitivity does not fall too far. In our hands, the ROC analysis supported the use of higher thresholds than did the other methods. Unfortunately the reference material was not sampled from any natural population and so the estimated sensitivity and specificity of the test based on ROC were unlikely to reflect the true performance in the populations that were sampled in this study [59].

We explored three internally calibrated thresholding methods (i.e. using only data generated during the study), all of which specified thresholds at approximately the same OD_450_ value. This was true across sample sets from all three countries. It is perhaps unsurprising that similar estimates emerged from FMM and EM, as there are methodological similarities in the two approaches. At face value the VIP method might seem arbitrary and crude, but the human brain can outperform computers in some aspects of pattern recognition and by obtaining a threshold estimate that closely matches that of EM and FMM, our data indicate that the results of a conditionally independent method (VIP) correlate closely with the computational approaches and are able to successfully determine where the most obvious bimodal split in the data occurs. What gives FMM and EM the edge over VIP is that they are more replicable and that the different requirements for higher or lower specificity and sensitivity in different clinical settings can be controlled by changing the number of SDs that the algorithm uses to determine the cut point. For instance, an increasingly specific test could be implemented by setting the threshold at four, five or six SDs of the negative population, rather than three SDs we used here. None of the populations that we surveyed would be expected (based on clinical signs) to have a high level of Ct seropositivity and it may be that the data in Tables 3 and 4 (and Supplementary Data S4, S5 and S6 Tables) reflect a high false positive rate, low positive predictive value. By adjusting the parameters of the algorithms we might achieve a prevalence estimate that is more accurate, but without any gold standard we can never truly assess how accurate our estimates are. In the Gambian data, using respectively 4 or 5 SDs would have led to cut points at respectively OD = 0.81 and OD = 0.95, values much closer to the cut-points recommended by the ROC analysis.

For programmatic purposes, the absolute value and accuracy of the prevalence estimate is actually somewhat less important than the precision of that estimate and the longitudinal change in repeat measures from the same population across the lifetime of the intervention and monitoring programme. This is because the absolute estimate is clearly highly variable given quite arbitrary choices made during data analysis, whilst percentage changes in population seroprevalence across time (regardless of the actual number values) can be indicative of the effectiveness of MDA. As long as the method is fixed and replicable, then both longitudinal and between–population comparisons are appropriate and will have a fixed level of error, even though the absolute accuracy will remain unknown. The real value of using an internally controlled method such as FMM or EM is that it is possible to use an algorithmic approach that is simple to apply to any data set and which requires no additional testing of external specimens or controls. In this study, we generated a ROC curve based on specimens that had previously been calibrated against the original reference standards described by Goodhew *et al* [19]. There is no gold standard for serological testing of chlamydia, and mis-classification in the reference standards is likely to have introduced error in the reference panel. Goodhew described how one PCR-positive DBS tested negative for antibodies against Pgp3, while three samples that were in the negative reference group tested positive for antibodies against Pgp3 [19]. As these original reference standards were no longer available, we have had to rely on a second set of standards that were tested against the original standards. Problems relating to the ROC reference specimens could be solved by the establishment of a fully maintained and quality controlled international standard, but this is unlikely to happen as it is would be very difficult to identify a reliable source of large volumes of seropositive plasma.

FMM has been used in numerous serological studies [34,39,43,45,60–66] and we propose that it, or the closely related EM, should be considered as the method of choice when performing data analysis for trachoma serology data. In trachoma control programmes, the SD parameter should be adjusted to favour high specificity and a larger number of SDs than used here would seem appropriate. One attractive option would be to use data from a post-elimination country (i.e. the Gambia) to subtract out the background positivity and by doing so calibrate or normalise the test for use in populations where elimination has not yet been reached and prevalence is unknown.

Variability and error are inherent to any diagnostic test and with every change in reference standard and assay technique, variability and error increase over and above any variation that may be inherent in a test due to inter- or intra- centre and user variation. Thus, we believe that an alternate approach to assay design, reference selection and threshold specification should be considered.

For all the sample sets included in this study, the density data peak around 0.25 OD_450_ (Figs 2A, 3A and 4A), suggesting that a comparison of seroprevalence levels between populations is possible. Compared to ROC curves, internally-referenced thresholds inherently account for differing background levels in each population. If not accounted for using the ROC curve, this may result in an under- or over-estimation of seroprevalence. This will facilitate the programmatic usage of seroprevalence levels set by the finite mixture model or expectation-maximisation algorithm if serology is to be adopted as an alternative monitoring method.

## Conclusion

The ELISA assay presented in this paper is easy-to-use, affordable in terms of both reagents and equipment required, and can potentially be deployed in low- and middle-income countries. The unit cost per sample was less than £4.00; this includes all materials required for sample collection and DBS testing, including reagents, ELISA plates and sterile gloves. Our results show that the technological aspects of the assay are robust and that there is low variation both between replicate samples and plates and between populations, making it possible to compare seroprevalence levels between countries. Internally calibrated thresholding methods, such as the finite mixture model or the expectation-maximisation algorithm are more appropriate than thresholds set by a ROC curve, but for programmatic surveillance, they may require calibration using data from countries where trachoma has been declared as having been eliminated.

## Supporting Information

S1 Table Prevalence of the clinical signs of trachoma for Laos, by Gender, Region and Age. (DOCX)

S2 Table Prevalence of the clinical signs of trachoma for Uganda, by Gender, Region and Age. (DOCX)

S3 Table Prevalence of the clinical signs of trachoma for The Gambia by Gender, Region and Age. (DOCX)

S4 Table Seroprevalence for Laos by Gender, Region and Age, for each of six thresholds. (DOCX)

S5 Table Seroprevalence for Uganda by Gender, Region and Age, for each of six thresholds. (DOCX)

S6 Table Seroprevalence for The Gambia by Gender, Region and Age, for each of six thresholds. (DOCX)

## References

[1] Beatty WL, Morrison RP, Byrne GI. Persistent chlamydiae: from cell culture to a paradigm for chlamydial pathogenesis. Microbiol Rev. 1994;58: 686–99. Available: http://www.pubmedcentral.nih.gov/articlerender.fcgi?artid=372987&tool=pmcentrez&rendertype=abstract 7854252 10.1128/mr.58.4.686-699.1994 PMC372987

[2] World Health Organisation. WHO alliance for the global elimination of Blinding trachoma by the year 2020. Wkly Epidemiol Rec. 2014;96: 421–428. 25275153

[3] World Health Organisation. WHO | Trachoma Fact sheet N 382 [Internet]. World Health Organization; 2016 [cited 12 Aug 2016]. Available: http://www.who.int/mediacentre/factsheets/fs382/en/

[4] Bourne RRA, Stevens GA, White RA, Smith JL, Flaxman SR, Price H, et al Causes of vision loss worldwide, 1990–2010: A systematic analysis. Lancet Glob Heal. 2013;1: 339–349. 10.1016/S2214-109X(13)70113-X 25104599

[5] Thylefors B, Dawson CR, Jones BR, West SK, Taylor HR. A simple system for the assessment of trachoma and its complications. Bull World Health Organ. 1987;65: 477–83. Available: http://www.pubmedcentral.nih.gov/articlerender.fcgi?artid=2491032&tool=pmcentrez&rendertype=abstract 3500800 PMC2491032

[6] Burton MJ, Holland MJ, Faal N, Aryee EA, Alexander N DE, Bah M, et al Which Members of a Community Need Antibiotics to Control Trachoma? Conjunctival Chlamydia trachomatis Infection Load in Gambian Villages. Invest Ophthalmol Vis Sci. 2003;44: 4215–4222. 14507864 10.1167/iovs.03-0107

[7] Harding-Esch EM, Sillah A, Edwards T, Burr SE, Hart JD, Joof H, et al Mass Treatment with Azithromycin for Trachoma: When Is One Round Enough? Results from the PRET Trial in The Gambia. Vinetz JM, editor. PLoS Negl Trop Dis. Public Library of Science; 2013;7: e2115 10.1371/journal.pntd.0002115 23785525 PMC3681669

[8] Burton MJ, Holland MJ, Makalo P, Aryee EAN, Alexander NDE, Sillah A, et al Re-emergence of Chlamydia trachomatis infection after mass antibiotic treatment of a trachoma-endemic Gambian community: a longitudinal study. Lancet. 2005;365: 1321–8. 10.1016/S0140-6736(05)61029-X 15823382

[9] Harding-Esch EM, Edwards T, Sillah A, Sarr I, Roberts CH, Snell P, et al Active trachoma and ocular Chlamydia trachomatis infection in two Gambian regions: on course for elimination by 2020? Ngondi JM, editor. PLoS Negl Trop Dis. Public Library of Science; 2009;3: e573 10.1371/journal.pntd.0000573 20027217 PMC2791206

[10] Burton MJ, Hu VH, Massae P, Burr SE, Chevallier C, Afwamba IA, et al What is causing active trachoma? The role of nonchlamydial bacterial pathogens in a low prevalence setting. Invest Ophthalmol Vis Sci. 2011;52: 6012–7. 10.1167/iovs.11-7326 21693601 PMC3176035

[11] Burr SE, Hart JD, Edwards T, Baldeh I, Bojang E, Harding-Esch EM, et al Association between ocular bacterial carriage and follicular trachoma following mass azithromycin distribution in The Gambia. Ngondi JM, editor. PLoS Negl Trop Dis. Public Library of Science; 2013;7: e2347 10.1371/journal.pntd.0002347 23936573 PMC3723595

[12] Roberts C h, Last A, Molina-Gonzalez S, Cassama E, Butcher R, Nabicassa M, et al Development and evaluation of a next generation digital PCR diagnostic assay for ocular Chlamydia trachomatis infections. J Clin Microbiol. 2013; 10.1128/JCM.00622-13 PMC3697714 23637300

[13] Jenson A, Dize L, Mkocha H, Munoz B, Lee J, Gaydos C, et al Field evaluation of the Cepheid GeneXpert Chlamydia trachomatis assay for detection of infection in a trachoma endemic community in Tanzania. Vinetz JM, editor. PLoS Negl Trop Dis. Public Library of Science; 2013;7: e2265 10.1371/journal.pntd.0002265 23861986 PMC3701699

[14] Dize L, West S, Williams JA, Van Der Pol B, Quinn TC, Gaydos CA. Comparison of the Abbott m2000 RealTime CT assay and the Cepheid GeneXpert CT/NG assay to the Roche Amplicor CT assay for detection of Chlamydia trachomatis in ocular samples from Tanzania. J Clin Microbiol. 2013;51: 1611–3. 10.1128/JCM.00519-13 23486714 PMC3647953

[15] Dize L, West S, Quinn TC, Gaydos CA. Pooling ocular swab specimens from Tanzania for testing by Roche Amplicor and Aptima Combo 2 assays for the detection of Chlamydia trachomatis: accuracy and cost-savings. Diagn Microbiol Infect Dis. NIH Public Access; 2013;77: 289–91. 10.1016/j.diagmicrobio.2013.08.005 24079951 PMC4160034

[16] Shekhawat N, Mkocha H, Munoz B, Gaydos C, Dize L, Quinn TC, et al Cohort and Age Effects of Mass Drug Administration on Prevalence of Trachoma: A Longitudinal Study in Rural Tanzania. Investig Opthalmology Vis Sci. 2014;55: 2307. 10.1167/iovs.13-12701 PMC3985515 24448262

[17] Harding-Esch E, Jofre-Bonet M, Dhanjal JK, Burr S, Edwards T, Holland M, et al Costs of Testing for Ocular Chlamydia trachomatis Infection Compared to Mass Drug Administration for Trachoma in The Gambia: Application of Results from the PRET Study. PLoS Negl Trop Dis. 2015;9: e0003670 10.1371/journal.pntd.0003670 25901349 PMC4406756

[18] Roberts CH, Last A, Burr SE, Bailey RL, Mabey DC, Holland MJ. Will droplet digital PCR become the test of choice for detecting and quantifying ocular Chlamydia trachomatis infection? Maybe. Expert Rev Mol Diagn. Informa UK, Ltd. London; 2014;14: 253–6. 10.1586/14737159.2014.897609 24649815

[19] Goodhew EB, Priest JW, Moss DM, Zhong G, Munoz B, Mkocha H, et al CT694 and pgp3 as Serological Tools for Monitoring Trachoma Programs. PLoS Negl Trop Dis. 2012;6: e1873 10.1371/journal.pntd.0001873 23133684 PMC3486877

[20] Goodhew EB, Morgan SMG, Switzer AJ, Munoz B, Dize L, Gaydos C, et al Longitudinal analysis of antibody responses to trachoma antigens before and after mass drug administration. BMC Infect Dis. England: BioMed Central Ltd.; 2014;14: 216 10.1186/1471-2334-14-216 24755001 PMC4016634

[21] Horner PJ, Wills GS, Reynolds R, Johnson AM, Muir D a, Winston A, et al Effect of time since exposure to Chlamydia trachomatis on chlamydia antibody detection in women: a cross-sectional study. Sex Transm Infect. England; 2013;89: 398–403. 10.1136/sextrans-2011-050386 23430706

[22] West SK, Munoz B, Weaver J, Mrango Z, Dize L, Gaydos C, et al Can We Use Antibodies to Chlamydia trachomatis as a Surveillance Tool for National Trachoma Control Programs? Results from a District Survey. PLoS Negl Trop Dis. 2016;10: 1–11. 10.1371/journal.pntd.0004352 PMC4714879 26771906

[23] Martin DL, Bid R, Sandi F, Goodhew EB, Massae PA, Lasway A, et al Serology for trachoma surveillance after cessation of mass drug administration. PLoS Negl Trop Dis. United States: Public Library of Science; 2015;9: e0003555 10.1371/journal.pntd.0003555 25714363 PMC4340913

[24] Pant BP, Bhatta RC, Chaudhary JSP, Awasthi S, Mishra S, Sharma S, et al Control of Trachoma from Achham District, Nepal: A Cross-Sectional Study from the Nepal National Trachoma Program. PLoS Negl Trop Dis. United States: Public Library of Science; 2016;10: e0004462 10.1371/journal.pntd.0004462 26871898 PMC4752456

[25] Wang J, Zhang Y, Lu C, Lei L, Yu P, Zhong G. A genome-wide profiling of the humoral immune response to Chlamydia trachomatis infection reveals vaccine candidate antigens expressed in humans. J Immunol. 2010;185: 1670–80. 10.4049/jimmunol.1001240 20581152

[26] Donati M, Laroucau K, Storni E, Mazzeo C, Magnino S, Di Francesco A, et al Serological response to pgp3 protein in animal and human chlamydial infections. Vet Microbiol. 2009;135: 181–185. 10.1016/j.vetmic.2008.09.037 18945555

[27] Galaleldeen A, Taylor AB, Chen D, Schuermann JP, Holloway SP, Hou S, et al Structure of the chlamydia trachomatis immunodominant antigen Pgp3. J Biol Chem. 2013;288: 22068–22079. 10.1074/jbc.M113.475012 23703617 PMC3724661

[28] Comanducci M, Cevenini R, Moroni A, Giuliani MM, Ricci S, Scarlato V, et al Expression of a plasmid gene of Chlamydia trachomatis encoding a novel 28 kDa antigen. J Gen Microbiol. 1993;139: 1083–92. Available: http://www.ncbi.nlm.nih.gov/pubmed/8336105 10.1099/00221287-139-5-1083 8336105

[29] Li Z, Zhong Y, Lei L, Wu Y, Wang S, Zhong G. Antibodies from women urogenitally infected with C. trachomatis predominantly recognized the plasmid protein pgp3 in a conformation-dependent manner. BMC Microbiol. 2008;8: 90 10.1186/1471-2180-8-90 18541036 PMC2432062

[30] Corran PH, Cook J, Lynch C, Leendertse H, Manjurano A, Griffin J, et al Dried blood spots as a source of anti-malarial antibodies for epidemiological studies. Malar J. 2008;7: 195 10.1186/1475-2875-7-195 18826573 PMC2567984

[31] Smit PW, Elliott I, Peeling RW, Mabey D, Newton PN. An overview of the clinical use of filter paper in the diagnosis of tropical diseases. Am J Trop Med Hyg. 2014;90: 195–210. 10.4269/ajtmh.13-0463 24366501 PMC3919219

[32] Smit PW, van der Vlis T, Mabey D, Changalucha J, Mngara J, Clark BD, et al The development and validation of dried blood spots for external quality assurance of syphilis serology. BMC Infect Dis. BMC Infectious Diseases; 2013;13: 102 10.1186/1471-2334-13-102 23442198 PMC3586363

[33] Parker SP, Cubitt WD. The use of the dried blood spot sample in epidemiological studies. J Clin Pathol. 1999;52: 633–9. Available: http://www.pubmedcentral.nih.gov/articlerender.fcgi?artid=501537&tool=pmcentrez&rendertype=abstract 10655983 10.1136/jcp.52.9.633 PMC501537

[34] Vyse AJ, Gay NJ, Hesketh LM, Pebody R, Morgan-Capner P, Miller E. Interpreting serological surveys using mixture models: the seroepidemiology of measles, mumps and rubella in England and Wales at the beginning of the 21st century. Epidemiol Infect. 2006;134: 1303–12. 10.1017/S0950268806006340 16650326 PMC2870519

[35] Solomon AW, Pavluck AL, Courtright P, Aboe A, Adamu L, Alemayehu W, et al The Global Trachoma Mapping Project: Methodology of a 34-Country Population-Based Study. Ophthalmic Epidemiol. 2015;22: 214–25. 10.3109/09286586.2015.1037401 26158580 PMC4687001

[36] Courtright P, Gass K, Lewallen S, MacArthur C, Pavluck A, Solomon AW, et al. Global Trachoma Mapping Project Training for mapping of trachoma, 3rd edition [Internet]. London; 2015. Available: http://www.trachomacoalition.org/resources/global-trachoma-mapping-project-training-mapping-trachoma

[37] Southisombath K, Sisalermsak S, Chansan P, Akkhavong K, Phommala S, Lewallen S, et al National Trachoma Assessment in the Lao People’s Democratic Republic in 2013–2014. Ophthalmic Epidemiol. Taylor & Francis; 2016;23: 1–7. 10.1080/09286586.2016.1236973 PMC5706970 27846362

[38] R Core Team. R: A Language and Environment for Statistical Computing. In: R Foundation for Statistical Computing 2014.

[39] Parker RA, Erdman DD, Anderson LJ. Use of mixture models in determining laboratory criterion for identification of seropositive individuals: application to parvovirus B19 serology. J Virol Methods. 1990;27: 135–144. 2156877 10.1016/0166-0934(90)90130-8

[40] Dempster APA, Laird NMN, Rubin DDB. Maximum likelihood from incomplete data via the EM algorithm. J R Stat Soc Ser B Methodol. 1977;39: 1–38.

[41] Youden WJ. Index for rating diagnostic tests. Cancer. 1950;3: 32–35. 15405679 10.1002/1097-0142(1950)3:1<32::aid-cncr2820030106>3.0.co;2-3

[42] Deb P. FMM: Stata module to estimate finite mixture models [Internet]. 2012. Available: http://econpapers.repec.org/software/bocbocode/s456895.htm

[43] Gay NJ, Vyse AJ, Enquselassie F, Nigatu W, Nokes DJ. Improving sensitivity of oral fluid testing in IgG prevalence studies: application of mixture models to a rubella antibody survey. Epidemiol Infect. 2003;130: 285–91. Available: http://www.pubmedcentral.nih.gov/articlerender.fcgi?artid=2869964&tool=pmcentrez&rendertype=abstract 12729197 10.1017/s0950268802008051 PMC2869964

[44] Kaneko A, Chaves LF, Taleo G, Kalkoa M, Isozumi R, Wickremasinghe R, et al Characteristic age distribution of Plasmodium vivax infections after malaria elimination on Aneityum Island, Vanuatu. Infect Immun. 2014;82: 243–52. 10.1128/IAI.00931-13 24166950 PMC3911855

[45] Sepúlveda N, Stresman G, White MT, Drakeley CJ. Current mathematical models for analyzing anti-malarial antibody data with an eye to malaria elimination and eradication. J Immunol Res. 2015;2015. 10.1155/2015/738030 PMC4684866 26770994

[46] Fraley C, Raferty AE. Model-based Clustering, Discriminant Analysis, and Density Estimation. J Am Stat Assoc. 2002;97: 611–631. Available: https://www.stat.washington.edu/raftery/Research/PDF/fraley2002.pdf

[47] Carstensen B, Plummer M, Laara E, Hills M. Epi: A Package for Statistical Analysis in Epidemiology. R package version 2.0 [Internet]. 2016. Available: http://cran.r-project.org/package=Epi

[48] Fisher RA. On the Interpretation of χ2 from Contingency Tables, and the Calculation of P. J R Stat Soc. 1922;85: 87–94.

[49] MacDonald PL, Gardner RC. Type I Error Rate Comparisons of Post Hoc Procedures for I j Chi-Square Tables. Educ Psychol Meas. 2000;60: 735–754.

[50] Simes R. An improved Bonferroni procedure for multiple tests of significance. Biometrika. 1986;73: 751–4.

[51] Lyytikainen E, Kaasila M, Koskela P, Lehtinen M, Patama T, Pukkala E, et al Chlamydia trachomatis seroprevalence atlas of Finland 1983–2003. Sex Transm Infect. England; 2008;84: 19–22. 10.1136/sti.2007.027409 17911135

[52] Horner P, Soldan K, Vieira SM, Wills GS, Woodhall SC, Pebody R, et al trachomatis C. Pgp3 antibody prevalence in young women in England, 1993–2010. Trotter CL, editor. PLoS One. United States: Public Library of Science; 2013;8: e72001 10.1371/journal.pone.0072001 23991024 PMC3749119

[53] Wills GS, Horner PJ, Reynolds R, Johnson AM, Muir D a, Brown DW, et al Pgp3 antibody enzyme-linked immunosorbent assay, a sensitive and specific assay for seroepidemiological analysis of Chlamydia trachomatis infection. Clin Vaccine Immunol. 2009;16: 835–43. 10.1128/CVI.00021-09 19357314 PMC2691054

[54] Comanducci M, Manetti R, Bini L, Santucci A, Pallini V, Cevenini R, et al Humoral immune response to plasmid protein pgp3 in patients with Chlamydia trachomatis infection. Infect Immun. 1994;62: 5491–7. Available: http://www.pubmedcentral.nih.gov/articlerender.fcgi?artid=303293&tool=pmcentrez&rendertype=abstract 7960130 10.1128/iai.62.12.5491-5497.1994 PMC303293

[55] Ghaem-Maghami S, Ratti G, Ghaem-Maghami M, Comanducci M, Hay PE, Bailey RL, et al Mucosal and systemic immune responses to plasmid protein pgp3 in patients with genital and ocular Chlamydia trachomatis infection. Clin Exp Immunol. 2003;132: 436–42. Available: http://www.pubmedcentral.nih.gov/articlerender.fcgi?artid=1808734&tool=pmcentrez&rendertype=abstract 10.1046/j.1365-2249.2003.02163.x 12780690 PMC1808734

[56] Polack S, Brooker S, Kuper H, Mariotti S, Mabey D, Foster A. Mapping the global distribution of trachoma. Bull World Health Organ. 2005;83: 913–919. Available: http://www.scielosp.org/scielo.php?script=sci_arttext&pid=S0042-96862005001200013&lang=es 16462983 PMC2626493

[57] Harding-Esch EM, Edwards T, Mkocha H, Munoz B, Holland MJ, Burr SE, et al Trachoma prevalence and associated risk factors in the Gambia and Tanzania: baseline results of a cluster randomised controlled trial. Schachter J, editor. PLoS Negl Trop Dis. Public Library of Science; 2010;4: e861 10.1371/journal.pntd.0000861 21072224 PMC2970530

[58] Harding-Esch EM, Edwards T, Sillah A, Sarr-Sissoho I, Aryee EA, Snell P, et al Risk factors for active trachoma in The Gambia. Trans R Soc Trop Med Hyg. 2008;102: 1255–62. 10.1016/j.trstmh.2008.04.022 18502459 PMC3836170

[59] Banoo S, Bell D, Bossuyt P, Herring A, Mabey D, Poole F, et al Evaluation of diagnostic tests for infectious diseases: general principles. Nat Rev Microbiol. Nature Publishing Group; 2006;4: S20–S32. 10.1038/nrmicro1570 17366684

[60] Pfeiffer RM, Gail MH, Brown LM. Probability of helicobacter pylori infection based on IgG levels and other covariates using a mixture model. J Epidemiol Biostat. 2000;5: 267–75. Available: http://www.ncbi.nlm.nih.gov/pubmed/11142602 11142602

[61] Gay NJ. Analysis of serological surveys using mixture models: application to a survey of parvovirus B19. Stat Med. 1996;15: 1567–73. 10.1002/(SICI)1097-0258(19960730)15:14<1567::AID-SIM289>3.0.CO;2-G 8855482

[62] Vyse AJ, Gay NJ, Hesketh LM, Morgan-Capner P, Miller E. Seroprevalence of antibody to varicella zoster virus in England and Wales in children and young adults. Epidemiol Infect. 2004;132: 1129–34. Available: http://www.pubmedcentral.nih.gov/articlerender.fcgi?artid=2870205&tool=pmcentrez&rendertype=abstract 15635971 10.1017/s0950268804003140 PMC2870205

[63] Bretscher MT, Supargiyono S, Wijayanti MA, Nugraheni D, Widyastuti AN, Lobo NF, et al Measurement of Plasmodium falciparum transmission intensity using serological cohort data from Indonesian schoolchildren. Malar J. 2013;12: 21 10.1186/1475-2875-12-21 23327665 PMC3605132

[64] Drakeley CJ, Corran PH, Coleman PG, Tongren JE, McDonald SLR, Carneiro I, et al Estimating medium- and long-term trends in malaria transmission by using serological markers of malaria exposure. Proc Natl Acad Sci U S A. 2005;102: 5108–13. 10.1073/pnas.0408725102 15792998 PMC555970

[65] Stewart L, Gosling R, Griffin J, Gesase S, Campo J, Hashim R, et al Rapid assessment of malaria transmission using age-specific sero-conversion rates. PLoS One. 2009;4: e6083 10.1371/journal.pone.0006083 19562032 PMC2698122

[66] van den Hoogen LL, Griffin JT, Cook J, Sepúlveda N, Corran P, Conway DJ, et al Serology describes a profile of declining malaria transmission in Farafenni, The Gambia. Malar J. BioMed Central; 2015;14: 416 10.1186/s12936-015-0939-1 26492873 PMC4618886

