# Supplementary material for: Defining Seropositivity Thresholds for Use in Trachoma Elimination Studies
Source: PLoS Negl Trop Dis. 2017 Jan 18;11(1):e0005230. doi: 10.1371/journal.pntd.0005230 (PMC5242428; doi:10.1371/journal.pntd.0005230)
Supplement: S1 Table — (DOCX) [file pntd.0005230.s001.docx]

**Supplementary Table 1: Prevalence of the clinical signs of trachoma for Laos, by Gender, Region and Age.**

|  | **Prevalence of clinical signs (%)** | |
| --- | --- | --- |
|  | **N** | **TF** |
| **Overall** | 952 | 15 (1.6) |
| Female | 423 | 9 (2.1) |
| Male | 529 | 6 (1.1) |
| Attapu | 406 | 11 (2.7) |
| Houaphan | 307 | 3 (1.0) |
| Phôngsali | 239 | 1 (0.4) |
| 1 year old | 78 | 2 (2.6) |
| 2 years old | 105 | 3 (2.9) |
| 3 years old | 101 | 5 (5.0) |
| 4 years old | 127 | 0 |
| 5 years old | 114 | 1 (0.9) |
| 6 years old | 100 | 2 (2.0) |
| 7 years old | 100 | 1 (1.0) |
| 8 years old | 99 | 0 |
| 9 years old | 128 | 1 (0.8) |

TF = trachomatous inflammation, follicular
