# Supplementary material for: Defining Seropositivity Thresholds for Use in Trachoma Elimination Studies
Source: PLoS Negl Trop Dis. 2017 Jan 18;11(1):e0005230. doi: 10.1371/journal.pntd.0005230 (PMC5242428; doi:10.1371/journal.pntd.0005230)
Supplement: S2 Table — (DOCX) [file pntd.0005230.s002.docx]

**Supplementary Table 2: Prevalence of the clinical signs of trachoma for Uganda, by Gender, Region and Age**

|  | **Prevalence of clinical signs (%)** | | |
| --- | --- | --- | --- |
|  | **N** | **TF** | **TI** |
| **Overall** | 2700 | 93 (3.4) | 8 (0.3) |
| Agogo | 1353 | 43 (3.2) | 1 (0.1) |
| Pader | 1347 | 50 (3.7) | 7 (0.5) |
| Female | 1351 | 48 (3.6) | 6 (0.4) |
| Male | 1349 | 45 (3.6) | 2 (0.1) |
| 1 year old | 242 | 11 (4.5) | 2 (0.8) |
| 2 years old | 353 | 17 (4.8) | 0 |
| 3 years old | 358 | 16 (4.5) | 3 (0.8) |
| 4 years old | 336 | 14 (4.2) | 1 (0.3) |
| 5 years old | 329 | 12 (3.6) | 1 (0.3) |
| 6 years old | 333 | 8 (2.4) | 0 |
| 7 years old | 264 | 6 (2.3) | 1 (0.4) |
| 8 years old | 235 | 3 (1.3) | 0 |
| 9 years old | 250 | 6 (2.4) | 0 |

TF = trachomatous inflammation, follicular; TI = trachomatous inflammation-intense
