# Supplementary material for: Defining Seropositivity Thresholds for Use in Trachoma Elimination Studies
Source: PLoS Negl Trop Dis. 2017 Jan 18;11(1):e0005230. doi: 10.1371/journal.pntd.0005230 (PMC5242428; doi:10.1371/journal.pntd.0005230)
Supplement: S3 Table — (DOCX) [file pntd.0005230.s003.docx]

**Supplementary Table 3: Prevalence of the clinical signs of trachoma for The Gambia by Gender, Region and Age**

|  | **Prevalence of clinical signs (%)** | | | | | |
| --- | --- | --- | --- | --- | --- | --- |
|  | **N** | **TF** | **TI** | **TS** | **TT** | **CO** |
| **Overall** | 1868 | 30 (1.6) | 4 (0.2) | 78 (4.2) | 8 (0.4) | 1 (0.1) |
| LRR | 1028 | 18 (1.8) | 4 (0.4) | 55 (5.4) | 7 (0.7) | 1 (0.1) |
| URR | 840 | 12 (1.4) | 0 | 23 (2.7) | 1 (0.1) | 0 |
| Female | 1080 | 10 (0.9) | 3 (0.3) | 52 (4.8) | 5 (0.5) | 1 (0.1) |
| Male | 788 | 20 (2.5) | 1 (0.1) | 26 (3.3) | 3 (0.4) | 0 |
| <1 year old | 36 | 0 | 0 | 0 | 0 | 0 |
| 1 year old | 65 | 1 (1.5) | 0 | 0 | 0 | 0 |
| 2 years old | 88 | 3 (3.4) | 0 | 2 (2.3) | 0 | 0 |
| 3 years old | 101 | 8 (7.9) | 0 | 1 (1.0) | 0 | 0 |
| 4 years old | 96 | 5 (5.2) | 0 | 1 (1.0) | 0 | 0 |
| 5 years old | 96 | 3 (3.1) | 0 | 1 (1.0) | 0 | 0 |
| 6 years old | 89 | 0 | 0 | 2 (2.2) | 0 | 0 |
| 7 years old | 77 | 2 (2.6) | 1 (1.3) | 0 | 0 | 0 |
| 8 years old | 78 | 1 (1.3) | 0 | 0 | 0 | 0 |
| 9 years old | 52 | 2 (3.8) | 1 (1.9) | 0 | 0 | 0 |
| 10-19 | 412 | 4 (1.0) | 1 (0.2) | 2 (0.5) | 0 | 0 |
| 20-29 | 191 | 0 | 0 | 1 (0.5) | 0 | 0 |
| 30-39 | 152 | 1 (0.7) | 1 (0.7) | 5 (3.3) | 0 | 0 |
| 40-49 | 99 | 0 | 0 | 5 (5.1) | 0 | 0 |
| 50-59 | 95 | 0 | 0 | 15 (15.8) | 2 (2.1) | 0 |
| 60+ | 141 | 0 | 0 | 43 (30.5) | 6 (4.3) | 1 (0.7) |
| 1-9 year olds -LRR | 383 | 14 (3.7) | 2 (0.5) | 1 (0.3) | 0 | 0 |
| 1-9 year olds -URR | 359 | 11 (3.1) | 0 | 6 (1.7) | 0 | 0 |
| ≥10 year olds-LRR | 645 | 4 (0.6) | 2 (0.3) | 54 (8.45) | 7 (1.1) | 1 (0.2) |
| ≥10 year olds-URR | 481 | 1 (0.2) | 0 | 17 (2.6) | 1 (0.2) | 0 |

TF = trachomatous inflammation, follicular; TI = trachomatous inflammation-intense; TS = trachomatous scarring; TT = trachomatous trichiasis; CO = corneal opacity
