# Supplementary material for: Defining Seropositivity Thresholds for Use in Trachoma Elimination Studies
Source: PLoS Negl Trop Dis. 2017 Jan 18;11(1):e0005230. doi: 10.1371/journal.pntd.0005230 (PMC5242428; doi:10.1371/journal.pntd.0005230)
Supplement: S4 Table — (DOCX) [file pntd.0005230.s004.docx]

**Supplementary table 4: Seroprevalence for Laos by Gender, Region and Age, for each of six thresholds.**

| Threshold, % (95% confidence interval) | | | | | | | | | | | | | |
| --- | --- | --- | --- | --- | --- | --- | --- | --- | --- | --- | --- | --- | --- |
|  |  | VIP | | EM | | FMM | | ROC Youden’s J-index | | ROC Sensitivity>80% | | ROC Specificity>98% | |
|  | N | OD=0.619 | | OD=0.650 | | OD=0.696 | | OD=0.870 | | OD=0.965 | | OD=1.951 | |
| **Overall** | 952 | 11.30% | (9.4-13.6) | 10.40% | (8.6-12.6) | 15.60% | (13.3-18.0) | 6.70% | (5.3-8.6) | 6.30% | (4.9-8.1) | 1.10% | (0.5-2.0) |
| Female | 423 | 10.40% | (7.7-13.8) | 9.90% | (7.3-13.3) | 15.60% | (12.3-19.5) | 6.90% | (4.7-9.8) | 6.40% | (4.3-9.3) | 0.70% | (0.2-2.2) |
| Male | 529 | 12.10% | (9.5-15.3) | 10. 8% | (8.3-13.8) | 15.50% | (12.6-18.9) | 6.60% | (4.7-9.2) | 6.20% | (4.4-8.7) | 1.30% | (0.6-2.8) |
| Attapu | 406 | 4.40% | (2.7-7.0) | 3.70% | (2.2-6.2) | 9.40% | (6.8-12.7) | 0.70% | (0.2-2.3) | 0.70% | (0.19-2.3) | 0% | (0-1.1) |
| Houaphan | 307 | 21.50% | (17.1-26.6) | 20.50% | (16.2-25.6) | 24.10% | (19.5-29.4) | 16.00% | (12.1-20.7) | 15.00% | (11.3-19.6) | 2.30% | (1.0-4.8) |
| Phôngsali | 239 | 10.00% | (6.7-14.7) | 8.80% | (5.7-13.3) | 15.10% | (10.9-20.4) | 5.00% | (2.7-8.8) | 4.60% | (2.4-8.3) | 1.30% | (0.3-3.9) |
| 1 year old | 78 | 10.20% | (4.8-19.7) | 10.20% | (4.8-19.7) | 10.20% | (4.8-19.7) | 9.00% | (4.0-18.2) | 9.00% | (4.0-18.2) | 2.60% | (0.4-9.8) |
| 2 years old | 105 | 8.60% | (4.2-16.1) | 8.60% | (4.2-16.1) | 14.30% | (8.5-22.7) | 6.70% | (3.0-13.7) | 6.70% | (3.0-13.7) | 0% | (0-4.3) |
| 3 years old | 101 | 9.90% | (5.1-17.9) | 8.90% | (4.4-16.7) | 12.90% | (7.3-21.4) | 6.90% | (3.1-14.2) | 6.90% | (3.1-14.2) | 2.00% | (0.3-7.7) |
| 4 years old | 127 | 13.40% | (8.2-20.8) | 12.60% | (7.6-19.9) | 15.70% | (10.1-23.5) | 9.40% | (5.2-16.3) | 9.40% | (5.2-16.3) | 0.80% | (0.04-5.0) |
| 5 years old | 114 | 6.10% | (2.7-12.7) | 4.40% | (1.6-10.4) | 8.80% | (4.5-15.9) | 2.60% | (0.7-8.1) | 2.60% | (0.7-8.1) | 0.90% | (0.04-5.5) |
| 6 years old | 100 | 12.00% | (6.6-20.4) | 11.00% | (5.9-19.2) | 18.00% | (11.3-27.2) | 6.00% | (2.5-13.1) | 6.00% | (2.5-13.1) | 3.00% | (0.8-9.2) |
| 7 years old | 100 | 8.00% | (3.8-15.6) | 8.00% | (3.8-15.6) | 13.00% | (7.4-21.6) | 6.00% | (2.5-13.1) | 6.00% | (2.5-13.1) | 0% | (0-4.6) |
| 8 years old | 99 | 15.20% | (9.0-24.1) | 12.10% | (6.7-20.6) | 24.20% | (16.4-34.1) | 8.10% | (3.8-15.8) | 7.10% | (3.1-14.5) | 1.00% | (0.-6.3) |
| 9 years old | 128 | 17.20% | (11.3-25.1) | 16.40% | (10.7-24.2) | 21.10% | (14.6-29.4) | 6.35 | (2.9-12.30 | 3.90% | (1.4-9.3) | 0 | (0-3.6) |

TF = trachomatous inflammation, follicular; TI = trachomatous inflammation-intense; TS = trachomatous scarring; TT = trachomatous trichiasis; CO = corneal opacity
VIP = visual inflection point; EM = expectation-maximisation algorithm; FMM = finite mixture model; OD = optical density, measured at 450 nm
