# Supplementary material for: Defining Seropositivity Thresholds for Use in Trachoma Elimination Studies
Source: PLoS Negl Trop Dis. 2017 Jan 18;11(1):e0005230. doi: 10.1371/journal.pntd.0005230 (PMC5242428; doi:10.1371/journal.pntd.0005230)
Supplement: S5 Table — (DOCX) [file pntd.0005230.s005.docx]

**Supplementary Table 5: Seroprevalence for Uganda by Gender, Region and Age, for each of six thresholds.**

| Threshold, % (95% confidence interval) | | | | | | | | | | | | | |
| --- | --- | --- | --- | --- | --- | --- | --- | --- | --- | --- | --- | --- | --- |
|  |  | VIP | | EM | | FMM | | ROC Youden’s J-index | | ROC Sensitivity>80% | | ROC Specificity>98% | |
|  | N | OD=0.641 | | OD=0.450 | | OD=0.554 | | OD=0.870 | | OD=0.965 | | OD=1.951 | |
| **Overall** | 2700 | 13.40% | (12.1-14.7) | 24.30% | (22.7-26.0) | 17.10% | (16.0-18.9) | 6.80% | (5.9-7.8) | 5.30% | (4.5-6.2) | 0.30% | (0.1-0.8) |
| Female | 1351 | 13.30% | (11.6-15.3) | 23.80% | (21.5-26.1) | 17.20% | (15.7-19.1) | 6.20% | (5.0-7.7) | 5.00% | (3.9-6.3) | 0.30% | (0.1-0.8) |
| Male | 1349 | 13.40% | (11.7-15.4) | 24.90% | (22.6-27.3) | 17.80% | (15.8-20.0) | 7.40% | (6.1-9.0) | 5.60% | (4.5-7.0) | 0.20% | (0.1-0.7) |
| Agogo | 1353 | 12.60% | (10.9-14.6) | 23.30% | (21.1-25.6) | 15.90% | (14.0-18.0) | 6.00% | (4.8-7.4) | 4.90% | (3.8-6.2) | 0.30% | (0.1-0.8) |
| Pader | 1347 | 14.10% | (12.3-16.1) | 25.40% | (23.1-27.8) | 18.90% | (16.9-21.1) | 7.70% | (6.3-9.2) | 5.70% | (4.6-7.1) | 0.20% | (0.1-0.7) |
| 1 year old | 247 | 2.40% | (1.0-5.5) | 7.30% | (4.5-11.4) | 3.60% | (1.8-7.0) | 1.60% | (0.5-4.4) | 0.80% | (0.1-3.2) | 0 | (0-1.9) |
| 2 years old | 365 | 5.50% | (3.5-8.5) | 10.70% | (7.8-14.4) | 7.90% | (5.5-11.3) | 3.80% | (2.2-6.5) | 3.80% | (2.2-6.5) | 0.30% | (0-1.8) |
| 3 years old | 367 | 7.10% | (4.8-10.3) | 18.30% | (14.5-22.7) | 10.60% | (7.8-14.3) | 3.50% | (2.0-6.1) | 2.20% | (1.0-4.4) | 0.30% | (0-1.8) |
| 4 years old | 349 | 11.50% | (8.4-15.4) | 22.10% | (17.9-26.9) | 15.20% | (11.7-19.5) | 4.90% | (3.0-7.8) | 4.00% | (2.3-6.8) | 0 | (0-1.4) |
| 5 years old | 331 | 15.70% | (12.1-20.2) | 27.20% | (22.5-32.4) | 18.40% | (14.5-23.1) | 8.20% | (5.5-11.8) | 6.60% | (4.3-10.0) | 0.60% | (0.1-2.0) |
| 6 years old | 344 | 18.30% | (14.5-22.9) | 31.10% | (26.3-36.3) | 23.50% | (19.2-28.5) | 8.10% | (5.6-11.7) | 6.40% | (4.1-9.7) | 0 | (0-1.4) |
| 7 years old | 269 | 16.70% | (12.6-21.9) | 32.30% | (26.9-38.3) | 23.40% | (18.6-29.0) | 9.30% | (6.2-13.6) | 6.70% | (4.1-10.5) | 0 | (0-1.8) |
| 8 years old | 240 | 20.40% | (15.6-26.2) | 32.50% | (26.7-38.9) | 24.20% | (19.0-30.2) | 9.20% | (6.0-13.7) | 7.50% | (4.6-11.8) | 0.80% | (0.1-3.3) |
| 9 years old | 253 | 23.70% | (18.7-29.5) | 37.20% | (31.2-43.4) | 30.40% | (24.9-36.6) | 13.40% | (9.6-18.4) | 9.90% | (6.6-14.4) | 0.40% | (0-2.5) |

TF = trachomatous inflammation, follicular; TI = trachomatous inflammation-intense; TS = trachomatous scarring; TT = trachomatous trichiasis; CO = corneal opacity
VIP = visual inflection point; EM = expectation-maximisation algorithm; FMM = finite mixture model; OD = optical density, measured at 450 nm
