# Supplementary material for: Defining Seropositivity Thresholds for Use in Trachoma Elimination Studies
Source: PLoS Negl Trop Dis. 2017 Jan 18;11(1):e0005230. doi: 10.1371/journal.pntd.0005230 (PMC5242428; doi:10.1371/journal.pntd.0005230)
Supplement: S6 Table — (DOCX) [file pntd.0005230.s006.docx]

**Supplementary Table 6: Seroprevalence for The Gambia by Gender, Region and Age, for each of six thresholds.**

| Threshold, % (95% confidence interval) | | | | | | | | | | | | | |
| --- | --- | --- | --- | --- | --- | --- | --- | --- | --- | --- | --- | --- | --- |
|  |  | VIP | | EM | | FMM | | ROC Youden’s J-index | | ROC Sensitivity>80% | | ROC Specificity>98% | |
|  | N | OD=0.570 | | OD=0.570 | | OD=0.672 | | OD=0.870 | | OD=0.965 | | OD=1.951 | |
| **Overall** | 1868 | 29.30% | (27.3-31.5) | 29.30% | (27.3-31.5) | 26.20% | (24.2-28.2) | 20.90% | (19.1-22.9) | 18.90% | (17.2-20.8) | 3.30% | (2.6-4.3) |
| Female | 1080 | 35.00% | (32.2-37.9) | 35.00% | (32.2-37.9) | 31.60% | (28.8-34.5) | 26.00% | (23.4-28.8) | 23.70% | (21.2-26.4) | 4.80% | (3.7-6.3) |
| Male | 788 | 21.60% | (18.8-24.6) | 21.60% | (18.8-24.6) | 18.70% | (16.1-21.7) | 14.00% | (11.7-16.6) | 12.30% | (10.1-14.9) | 1.30% | (0.7-2.4) |
| LRR | 1028 | 33.90% | (31.0-36.8) | 33.80% | (31.0-36.8) | 30.50% | (27.7-33.4) | 25.10% | (22.5-27.9) | 22.60% | (20.1-25.2) | 3.70% | (2.7-5.1) |
| URR | 840 | 23.80% | (21.0-26.9) | 23.80% | (21.0-26.9) | 21.00% | (18.3-23.9) | 15.80% | (13.5-18.5) | 14.40% | (12.1-17.0) | 2.90% | (1.9-4.3) |
| <1 year | 36 | 0 | (0-12.0) | 0 | (0-12.0) | 0 | (0-12.0) | 0 | (0-12.0) | 0.00 | (0-12.0) | 0.00 | (0-12.0) |
| 1 year old | 65 | 4.60% | (1.2-13.8) | 4.60% | (1.2-13.8) | 3.10% | (0.5-11.6) | 0 | (0-6.9) | 0 | (0-6.9) | 0 | (0-6.9) |
| 2 years old | 88 | 4.50% | (1.5-11.9) | 4.50% | (1.5-11.9) | 2.30% | (0.4-8.7) | 1.10% | (0-7.1) | 0 | (0-5.2) | 0 | (0-5.2) |
| 3 years old | 101 | 5.90% | (2.4-13.0) | 5.90% | (2.4-13.0) | 5.90% | (2.4-13.0) | 2.00% | (0.3-7.7) | 2.00% | (0.3-7.7) | 0 | (0-4.6) |
| 4 years old | 96 | 8.30% | (3.9-16.2) | 8.30% | (3.9-16.2) | 7.30% | (3.2-14.90 | 4.20% | (1.3-10.9) | 4.20% | (1.3-10.9) | 0 | (0-4.8) |
| 5 years old | 96 | 8.30% | (3.9-16.2) | 8.30% | (3.9-16.2) | 6.30% | (2.6-13.6) | 3.10% | (0.8-9.5) | 3.10% | (0.8-9.5) | 0 | (0-4.8) |
| 6 years old | 89 | 5.60% | (2.1-13.2) | 5.60% | (2.1-13.2) | 4.50% | (1.4-11.7) | 4.50% | (1.4-11.7) | 3.40% | (0.9-10.2) | 0 | (0-5.2) |
| 7 years old | 77 | 10.40% | (4.9-20.0) | 10.40% | (4.9-20.0) | 7.80% | (3.2-16.8) | 2.60% | (0.5-9.9) | 2.60% | (0.5-9.9) | 0 | (0-5.9) |
| 8 years old | 78 | 10.30% | (4.8-19.7) | 10.30% | (4.8-19.7) | 7.70% | (3.2-16.6) | 6.40% | (2.4-15.0) | 3.80% | (1.0-11.6) | 0 | (0-5.8) |
| 9 years old | 52 | 19.20% | (10.1-33.0) | 19.20% | (10.1-33.0) | 15.40% | (7.3-28.6) | 9.60% | (3.6-21.8) | 9.60% | (3.6-21.8) | 1.90% | (0.1-11.6) |
| 10-19 | 412 | 21.40% | (17.6-25.7) | 21.40% | (17.6-25.7) | 15.50% | (12.2-19.5) | 11.10% | (8.4-14.7) | 9.70% | (7.1-13.1) | 1.70% | (0.7-3.6) |
| 20-29 | 191 | 36.60% | (29.9-43.9) | 36.60% | (29.9-43.9) | 31.40% | (25.0-38.6) | 23.00% | (17.4-29.8) | 19.90% | (14.6-26.4) | 3.70% | (1.6-7.7) |
| 30-39 | 152 | 53.90% | (45.7-62.0) | 53.90% | (45.7-62.0) | 52.00% | (43.8-60.1) | 46.10% | (38.0-54.3) | 44.10% | (36.1-52.3) | 5.90% | (2.9-11.3) |
| 40-49 | 99 | 69.70% | (59.5-78.3) | 69.70% | (59.5-78.3) | 65.70% | (55.4-74.7) | 56.60% | (46.2-66.4) | 50.10% | (40.3-60.1) | 12.10% | (6.7-20.6) |
| 50-59 | 95 | 74.70% | (64.5-82.8) | 74.70% | (64.5-82.8) | 72.60% | (62.4-81.0) | 62.10% | (51.5-71.7) | 55.80% | (45.3-65.9) | 8.40% | (4.0-16.4) |
| 60+ | 141 | 75.90% | (67.8-82.5) | 75.90% | (67.8-82.5) | 73.80% | (65.6-80.6) | 63.10% | (54.5-71.0) | 58.20% | (49.5-66.3) | 12.80% | (7.9-19.7) |

TF = trachomatous inflammation, follicular; TI = trachomatous inflammation-intense; TS = trachomatous scarring; TT = trachomatous trichiasis; CO = corneal opacity
VIP = visual inflection point; EM = expectation-maximisation algorithm; FMM = finite mixture model; OD = optical density, measured at 450nm
